# Emotional Modulation of Episodic Memory in School-Age Children and Adults: An Event-Related Potential Study

**DOI:** 10.3390/brainsci11121598

**Published:** 2021-11-30

**Authors:** Sarah Massol, Cora Caron, Nicolas Franck, Caroline Demily, Hanna Chainay

**Affiliations:** 1 Laboratoire d’Etude des Mécanismes Cognitifs (EMC), EA3082 Université Lumière Lyon, 69676 Bron, France; sarah.massol@univ-lyon2.fr; 2 Pôle Centre Rive Gauche et Centre Ressource de Réhabilitation Psychosociale, Centre Hospitalier le Vinatier et Institut Marc Jeannerod, UMR 5229 (CNRS et Université Claude Bernard Lyon 1), 69676 Bron, France; coracaron@gmail.com (C.C.); nicolas.franck@ch-le-vinatier.fr (N.F.); 3 Pôle Hospitalo-Universitaire ADIS, Centre de Référence Maladie Rares GénoPsy, Centre Hospitalier Le Vinatier et Institut Marc Jeannerod, UMR 5229 (CNRS et Université Claude Bernard Lyon 1), 69676 Bron, France; caroline.demily@ch-le-vinatier.fr

**Keywords:** school-age children, adults, emotions, episodic memory, ERPs

## Abstract

The Emotional Enhancement of Memory (EEM) has been well-demonstrated in adults, but less is known about EEM in children. The present study tested the impact of emotional valence of pictures on episodic memory using behavioral and neurophysiological measures. Twenty-six 8- to 11-year-old children were tested and compared to 30 young adults. Both groups participated in pictures’ intentional encoding tasks while event-related potentials (ERPs) were recorded, followed by immediate free recall tasks. Behavioral results revealed a general EEM in free recall performances in both groups, along with a *negativity effect* in children. ERP responses revealed a particular sensitivity to negative pictures in children with a late *emotion effect* at anterior clusters, as well as a greater *successful encoding effect* for emotional pictures compared to neutral ones. For adults, the *emotion effect* was more pronounced for positive pictures across all time windows from the centro-parietal to the frontal part, and localized in the left hemisphere. Positive pictures also elicited a greater *successful encoding effect* at anterior clusters in adults. By combining behavioral and neurophysiological measures to assess the EEM in children compared with adults, our study provides new knowledge concerning the interaction between emotional and memory processes during development.

## 1. Introduction

The emotional enhancement of memory (EEM), or having a better memory for emotional than neutral information, has been extensively demonstrated in adults using various paradigms with different types of information (pictures, words, stories, etc.) or memory tasks (for reviews, see [1,2]). Enhancing effects of emotion are notably attributed to two dimensions of emotional information: arousal, ranging from calm to exciting; and valence, ranging from unpleasant to pleasant [3,4,5,6]. Consistent with the cognitive mediation hypothesis, several psychological studies have suggested that the effects of emotion on memory can be partly explained by cognitive factors, such as increased attention toward emotional stimuli [7]. In this respect, emotional stimuli capture more attentional resources than neutral ones and are, therefore, more efficiently encoded in memory [8].

Event-related potential (ERP) studies provide a valuable opportunity to investigate this cognitive mediation of EEM and have been widely used in adults [8,9,10]. In particular, to better understand the EEM, the late positive potential (LPP), thought mainly to index greater attentional engagement toward emotional stimuli, was examined in these studies. The LPP can appear at several latency ranges (often categorized as early, middle, and late) mainly between 400 and 2000 ms after stimulus onset, and is widely distributed across the scalp with maximal voltage at the centro-parietal sites. The LPP-indexed attention modulation can reflect both an *emotion effect*, that is, a greater amplitude during the presentation of negative and positive stimuli compared to neutrals, and a greater *Dm effect* (or *subsequent memory effect*), that is, the differential brain activity during encoding associated with later remembered vs. forgotten stimuli, for emotional stimuli compared to neutral ones. Specifically, the Ref. [9] analyzed both of these effects and observed larger LPP during the presentation of emotional pictures compared to neutral ones at fronto-central and parietal locations (*emotion effect*), as well as a larger *Dm effect* for emotional pictures compared to neutral ones during the 400–600 ms time window, maximal at the midline central electrode. Similar results were found by the Ref. [10], with emotional pictures eliciting a larger *Dm effect* (during the 400–1500 ms time window at anterior and posterior sites) than neutral ones. Thus, LPP can index the EEM during encoding, suggesting that attentional engagement toward emotional stimuli is a reliable predictor of the EEM, even though some ERP modulations may vary depending on stimulus materials and experimental paradigms (for a review, see [11]).

Both behavioral and neural responses are modulated by age and the emotional valence of information. Some behavioral studies suggest that young adults have better memory for negative stimuli, while older adults have better memory for positive stimuli, which has been called the *negativity* and *positivity* effects, respectively ([12], for a review see [13]). These behavioral findings were also found at the neural level with several studies showing a modulation of the *emotion effect* with age, as reflected by LPP modulation [14,15,16]. These differences may be due to age-related differences in the availability of cognitive resources (e.g., [17]) or to differences in emotional regulation (e.g., [18]). This point is also important with regard to development. For example, children, like older adults, present specific patterns in the availability of cognitive resources and emotion processing in comparison to young adults (e.g., [19,20,21,22]). This is possibly in relation with the significant behavioral and cerebral development of emotional responses and regulation during childhood and adolescence (for a review, see [23]).

Studies investigating the EEM in children using various stimuli (e.g., pictures, words, stories) and various memory tasks (e.g., free recall, recognition) have shown either an EEM with better memory for both negative and positive stimuli alike in 6- to 11-year-old children [24], better memory for negative and positive stimuli with an advantage for negative ones in 5- to 6-year-old [25] and 8- to 11-year-old children [26], and better memory for negative compared to neutral stimuli without comparison with positive ones [27]. However, some studies have shown no effect of emotion on memory in children ([28,29], for a review, see [30]). Because all the studies did not assess the children’s EEM in relation to that in adults, it is not clear whether the EEM is consistent across development. An early hint has been given by [31] who investigated the effect of emotion on recognition memory across middle childhood, adolescence, and early adulthood in 8- to 30-year-old individuals. They showed similar results across the age range, with significantly better memory for negative and positive pictures relative to neutrals, showing great consistency in EEM from middle childhood to adulthood. Moreover, in this study, the negative-neutral memory difference was larger than the positive-neutral difference, suggesting a general EEM along with a *negativity* effect for all the participants. The study by the Ref. [26] also added evidence for this consistency across age with similar results obtained between groups of 8- to 11-year-old children and groups of young adults for the effects of emotion on item and associative memory. These behavioral findings should be further addressed through physiological measures to examine developmental changes in the neural correlates underlying emotional memory. Nevertheless, a few ERP studies have investigated emotional responses in children, and have shown that children as young as 5 years old elicit an adult-like *emotion effect* with an enhancement of the LPP in response to negative and positive pictures compared to neutral ones, in the middle and late time windows (from 700 to 2000 ms after stimulus onset) at the centro-parietal sites [32,33]. However, developmental changes seem to occur at the neural level with the scalp distribution of the LPP that shift from the occipital sites in middle childhood to more anterior regions in adolescence [19,34,35], associated with a decrease in amplitude until adulthood during the 400–1000 ms time window [36]. These changes in scalp distribution of the LPP suggest the contribution of different neural resources, and hence, cognitive processes, for the emotion processing across the development. In addition, to date, only two ERP studies have investigated the neural responses associated with the EEM in children [29,37]. The first authors have shown, despite the lack of emotional influence on recognition performance, a larger LPP during encoding and recognition of negative pictures in 7.5- to 9-year-old children relative to positive and neutral pictures. Moreover, the Ref. [37] analyzed the EEM separately for negative and positive stimuli across two recognition tasks in 8-year-old girls and showed enhanced recognition for negative, relative to neutral stimuli, but no difference in the recognition of positive and neutral stimuli. In ERP responses, however, the girls elicited larger LPP during the encoding and recognition of both negative and positive stimuli relative to neutral ones. Unfortunately, these studies did not compare the groups of children with adults, thus providing only a partial insight into the developmental neural changes associated with the EEM.

Therefore, in an attempt to further address the emotional modulation of episodic memory by age at both a behavioral and neural level, the present study investigated this phenomenon in 8- to 11-year-old children and young adults, using behavioral and ERP measures. This age limit was chosen for children with regard to a previous ERP study that showed more consistency between behavioral and ERP responses for the EEM from the age of 8 years [37]. More specifically, in the present study, the LPP was examined to provide some indications on the involvement of attentional mechanisms in the EEM in individuals who are still in a period of development and, therefore, to examine the relevance of the cognitive mediation hypothesis for this population. To this end, children and young adults participated in pictures’ intentional encoding tasks while ERPs were recorded, followed by immediate free-recall tasks. Recall tasks were used to better balance the number of remembered items with the number of forgotten items in order to correctly analyze the *Dm effect*. Concerning the recall performances, we hypothesized that negative and positive pictures would be better recalled than neutral ones in children and adults. Moreover, based on the idea that additional attention during the encoding of emotional stimuli is the main determinant of the EEM, we hypothesized that the LPP described above would be enhanced during the encoding of emotional, negative and positive, pictures relative to neutral ones (*emotion effect*) in both groups. In addition, as a *negativity* effect has often been demonstrated in children and adults for both behavioral and physiological responses, we also expected negative pictures to elicit greater free-recall performances and LPP amplitudes relative to positive ones in both groups. An additional analysis of the LPP during the successful encoding of pictures was performed to measure the *Dm effect* in both children and adults, as to observe the link between recall performance and ERPs. We expected a larger *Dm effect* for negative and positive pictures compared to neutral ones in both groups. Finally, some uncertainties remain regarding the scalp distribution of the ERPs in children, as 8- to 11-year-old children are still in a period of emotional development [19,34]. Thus, the evaluation of the ERP topography in children in relation to their recall performances will provide further knowledge about the development of the neural correlates underlying EEM.

## 2. Method

### 2.1. Participants

Twenty-six typically developing children (10 girls and 16 boys, 8–11 years of age, M = 9.83, SD = 1.45) and 30 young adults (16 women and 14 men, 18–28 years of age, M = 21.47, SD = 2.47) participated in this study. Our target sample size was determined using an a priori power analysis [38] via G*Power, using *η*²_p_ from previous studies investigating the effects of emotion on memory in children with recall tasks. For example, previous studies by the Refs. [25,26] found *η*²_p_ of 0.36 and 0.23, respectively. Using the lowest *η*²_p_ = 0.23, our study design with one between-participants factor (children and adults group) and one repeated factor—valence (negative, positive, neutral) could achieve with α = 0.05 80% power with 15 participants per group. In order to strengthen our study, we decided to include 30 participants per group, but succeeded in doing so only for the group of adults due to the health situation related to COVID-19 which interrupted the inclusion of additional children. All the participants were included before the pandemic. All participants were right-handed, as assessed by the Edinburgh Handedness Inventory [39]. Participants’ characteristics are presented in Table 1. Children were recruited from advertising and local elementary schools, and young adults were undergraduate students recruited at the university. All the participants were healthy French speakers, and reported no history of psychiatric, neurodevelopmental, neurological, or learning disorders. They were not taking any somatic medications and did not have any uncorrected visual problems. Prior to testing, the children’s parents and young adults gave informed consent, and the children gave verbal assent in accordance with the Helsinki declaration. The children received a €20 voucher and the adults were paid €20 for their participation in the study. The research was approved by the Ethical Review Board (Comité de Protection des Personnes Nord-Ouest IV, Lille, France, Nr. 2017 A03321 52).

### 2.2. Stimuli and Material

A total of 180 color photographs (567 × 425 pixels) depicting various types of scenes with common living and non-living elements (e.g., landscapes, animals, accidents, weapons) were used for each group of children and adults. The 60 positive, 60 negative, and 60 neutral pictures were selected for children and for adults from the Developmental Affective Photo System (DAPS: [27]), which provides emotional valence, arousal, and complexity ratings for pictures from children and adults. Among those pictures, 41 positive, 44 negative, and 43 neutral pictures were identical between children and adults. The remaining pictures were not exactly of the same content, and this was in order to ensure the level of valence and arousal to be the most similar for the negative, positive, and neutral pictures selected for the present study between adults and children (see Appendix A—Table A1). In fact, for some pictures in the DAPS, children’s and adults’ ratings differed too much.

The 180 pictures in each pool were split into six sets of 30 pictures (10 positive, 10 negative, and 10 neutral), which were assigned to six study-test blocks for both groups. Within each set and for both children and adults, the positive, negative, and neutral pictures significantly differed in terms of valence (all *p* < 0.0001) and the emotional pictures (negative and positive) were significantly more arousing than the neutral pictures (both *p* < 0.0001). The negative and positive pictures did not differ in terms of arousal (all *p* > 0.66). The positive, negative, and neutral pictures were also matched on visual complexity (all *p* > 0.28). See Appendix A—Table A1 for mean pictures’ ratings and standard deviations for children and adults.

### 2.3. Mood and Neuropsychological Assessments

The Face Mood Assessment Test for Children (FMATC—[40]) and the French version of the Brief Mood Introspection Scale (BMIS—[41]) were administered to self-report children’s and adults’ emotional states. For the FMATC, children had to select one of the six faces illustrating emotional expressions on a scale from 1 (sad) to 6 (happy). The BMIS is a mood adjective scale consisting of 16 mood adjectives which can identify, among other things, a pleasant/unpleasant mood. The BMIS was shortened to 12 mood adjectives for children by removing all the not commonly used adjectives that could have impeded understanding. Thus, the results ranged from 16 (very unpleasant mood) to 64 (very pleasant mood) for adults, and from 12 to 48 for children, respectively. In order to ensure that all children presented cognitive functioning within the normal range, the Wechsler Intelligence Scale for Children (WISC-V—[42]) was also administered to children to assess their verbal comprehension (similarities subtest) and their fluid reasoning (matrix reasoning subtest).

### 2.4. Procedure

**Figure 1:**
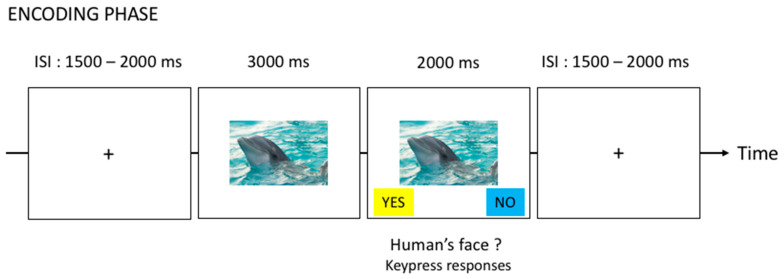
Procedure for encoding phase. The picture used for the illustrative purpose is a free-access picture downloaded from the website unsplash.com, (accessed on 25 October 2021).

Before starting the experiment, the participants completed the FMATC and BMIS to self-report on their emotional state. Then, they were explained that they would see six blocks of emotional and neutral pictures. Six different block orders were employed, and pictures within each block were presented successively in a pseudorandom order in such a way that no more than two pictures of the same valence occurred in succession for each participant. During the encoding phase of each block, the participants were asked to remember the pictures for the subsequent recall test, and to respond to the question, “Do you see a human face or part of a human face in the picture?” for each picture by pressing one of two keys corresponding to “yes” or “no”, respectively, and labeled with colored stickers. This was done to focus the participants’ attention on the pictures. The proportion of pictures that contained a human face or not was equivalent for each category of valence. Each picture was presented for a total of 5000 ms on a computer screen with the picture alone for the first 3000 ms and for an additional 2000 ms with the two options “yes” or “no” that appeared underneath the picture with the same colors as the corresponding computer responses. The participants were instructed to pay attention to the pictures and to wait until the two options appeared on the screen before making a keypress response to indicate if the picture contained a human face. The two computer responses were counterbalanced across participants. A jittered 1500–2000 ms inter-stimulus interval (ISI) was employed to prevent the habituation and development of picture onset expectations. During this interval, a central fixation cross was presented to ensure that the participants fixated on the center of the screen before picture onset (see Figure 1). After the encoding phase of each block, the participants completed a 1 min arithmetic task before starting the recall of pictures (test phase). Two simple sums were presented on the screen and the participants had to compute the sums mentally and identify the highest value sum by pressing one of two keys. For each test phase, the participants were asked to orally recall as many pictures as they could remember from the encoding phase (out of the 30) regardless of the order in which they had appeared, for 5 min. The participants were instructed to give as many details about the pictures as possible so that anyone could recognize them. If the experimenter felt that the description of the pictures was not precise enough, she asked the participant for clarification. The experimenter wrote down the participants’ answers. If there was any doubt about the identity of the picture during scoring, double-scoring was performed by one of the co-authors.

EEG was only recorded during the encoding phase of the pictures. The participants were told to remain still, stare at the center of the screen, and refrain from blinking as much as possible while the stimuli were displayed. The experimenter was looking at the continuous EEG data during recording, and feedback was given to the participants if they were not conforming to the instructions. All the participants followed the instructions well.

Practice trials with 15 neutral pictures for the encoding phase were presented at the beginning to make sure that the participants understood how to complete the task with the EEG instructions. This was repeated if necessary until it was clear that the participants understood and performed as instructed. The experiment was programmed and run with the E-Prime software (2.0) on a Dell PC computer.

At the end of the experiment, and after a break, two neuropsychological tests (described above) were administered to the children, in order to ensure that they all presented cognitive functioning within a normal range. All the children performed within the normal range. The raw and standard means performances for all the tests are presented in Table 1.

#### 2.4.1. ERP Data Recording and Reduction

The EEG was recorded from 128 Ag/AgCl electrode elements embedded in a cap (WaveGuard, ANT, Enschede, The Netherlands), conforming to the 10–20 international electrode placement system. The EEG signal was recorded using ASAlab 4.7 software. Impedances were under 5 kΩ and the sampling rate was set at 1024 Hz. Data were preprocessed using EEGlab v14.1.2 operating in Matlab R2018b. Data were referenced offline to the combined P9 and P10 electrodes, which are closest to the mastoids and thought to optimize LPP effects [43], filtered using a bandpass filter between 0.1 and 30 Hz, downsampled to 256 Hz, segmented into 2200 ms epochs (from 200 ms before to 2000 ms after picture onset), and baseline-corrected (200 ms prior to picture onset). Trials that were contaminated by positive or negative deflections that exceeded 50 µV were excluded from the analysis, using the 200 ms moving window peak-to-peak threshold for the automatic artifact detection. Data from six children and four adults were excluded due to poor quality EEG recording (*n* = 3 children and 1 adult) or due to more than 20 outliers in the data, seen through box plots in the descriptive statistics (*n* = 3 children and 3 adults). Thus, the final sample for the ERP data analysis included 20 children (8 girls and 12 boys, 8–11 years of age, M = 10.13, SD = 1.22) and 26 young adults (15 women and 11 men, 18–28 years of age, M = 21.75, SD = 2.46). To investigate the *emotion effect*, artifact-free epochs were averaged in three conditions: negative, positive, and neutral. This was done separately for children and adults. The mean numbers of epochs included in the ERP averages were: for children, M = 52.85 (5.69) epochs for negative, M = 51.40 (6.57) epochs for positive, and M = 51.45 (6.08) epochs for neutral conditions; and for adults, M = 58.00 (2.00) epochs for negative, M = 57.92 (1.67) epochs for positive, and M = 56.42 (2.34) epochs for neutral conditions. Additional analyses of the *successful encoding effect* (i.e., pictures that were later remembered) and the Difference Wave (DW) scores (i.e., pictures that were later remembered minus pictures that were later forgotten, called the *Dm effect*) in each condition were conducted on a subsample of participants who had more than 10 artifact-free epochs in each condition: 16 children (7 girls and 9 boys, 8–11 years of age, M = 10.20, SD = 1.29) and the same group of 26 young adults as above). The mean numbers of epochs included in the ERP averages for the successful encoding of pictures and the *Dm effect* are presented in Table 2 for both children and adults.

#### 2.4.2. ERP Data Analysis

**Figure 2:**
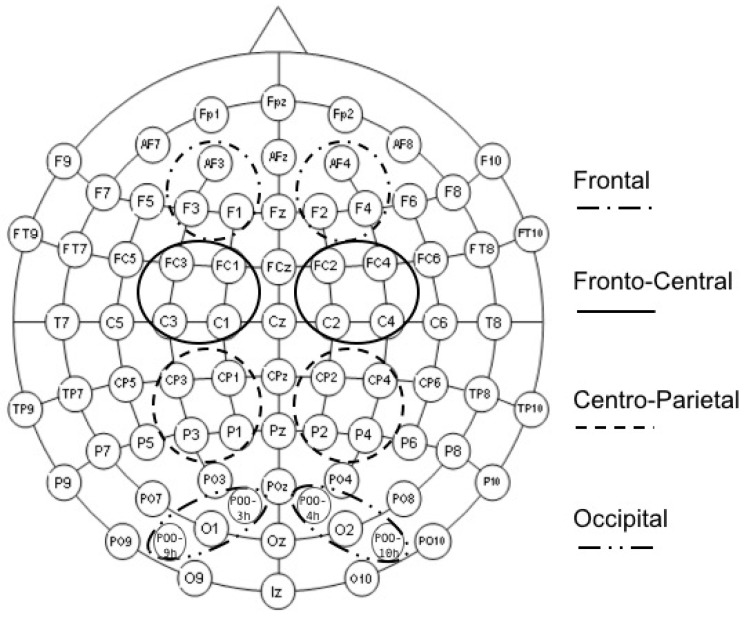
Electrode layout conforming to the 10–20 international system. The four clusters, separated into right and left hemisphere, are circled with different continuous or broken lines.

Averaged data were examined in light of previous studies [9,29,32,33,37] and, in particular, the shape of the encoding ERP waveforms revealed a sustained positive slow-wave consistent with the LPP over all electrodes. The LPP is interpreted for deflections toward positive amplitudes regardless of value. That is, only positive deflections were interpreted even if the effects at the frontal and central sites were negative. This is consistent with the approach taken by the Refs. [29,32,33,37]. For the six encoding blocks, the mean amplitude of the LPP was examined over five time windows (W1: 400–600 ms, W2: 600–800 ms, W3: 800–1200 ms, W4: 1200–1600 ms and W5: 1600–2000 ms) and four clusters of electrodes divided into right and left hemispheres (Frontal: AF3, AF4, F1, F2, F3, F4; Fronto-Central: FC1, FC2, FC3, FC4, C1, C2, C3, C4; Centro-Parietal: CP1, CP2, CP3, CP4, P1, P2, P3, P4; Occipital: O1, O2, POO3h, POO4h, POO9h, POO10h; clusters are depicted in Figure 2). These locations and time windows were selected based on previous studies on both the *emotion effect* and *Dm effect* [9,29,32,33,37] and a careful visual inspection of our own data in order to investigate the duration, timing, and topography of emotional and memory responses.

#### 2.4.3. Behavioral Statistical Analysis

The scores for correct free recall for the positive, negative, and neutral pictures were used for the statistical analysis. Preliminary analyses were performed to check for sphericity (Mauchly’s test) and homogeneity of variance (Levene’s test), with no violations being found for any of the data. A mixed-measures ANOVA was performed on the scores with Valence (negative vs. positive vs. neutral) as a within-subject factor and Group (children vs. adults) as a between-subject factor. We also included the within-subject factor Block to check the recall performances according to the blocks. These analyses were followed by post hoc comparisons with Bonferroni corrections.

#### 2.4.4. ERP Statistical Analysis

In order to investigate the *emotion effect*, the mean amplitudes of the averaged LPP waveforms were analyzed using mixed-measures ANOVAs. Preliminary analyses were performed to check for sphericity (Mauchly’s test). When violations were found, the Greenhouse-Geisser sphericity correction was applied to the data. First, we performed a global analysis using a mixed-measures ANOVA with Valence (negative vs. positive vs. neutral), Window (W1 vs. W2 vs. W3 vs. W4 vs. W5), Cluster (frontal vs. fronto-central vs. centro-parietal vs. occipital), and Hemisphere (right vs. left) as within-subject factors, and Group (children vs. adults) as a between-subject factor. Moreover, in order to investigate the *successful encoding effect*, we performed the same analysis as above but with the inclusion of the within-subject factor Recall (recalled pictures vs. forgotten pictures). Then, to more precisely identify these effects, we performed separate mixed-measures ANOVAs with the same factors as the first analyses but separately for children and adults. All these analyses were followed by post hoc comparisons with Bonferroni corrections.

In order to investigate the *Dm effect*, the mean amplitude of the averaged DW (i.e., pictures that were later remembered minus pictures that were later forgotten) was analyzed using mixed-measures ANOVAs. As described above, the same preliminary analyses were performed and the same corrections were applied if necessary. Thus, a mixed-measures ANOVA with Memory Valence (negative recalled minus negative forgotten vs. positive recalled minus positive forgotten vs. neutral recalled minus neutral forgotten), Window (W1 vs. W2 vs. W3 vs. W4 vs. W5), Cluster (frontal vs. fronto-central vs. centro-parietal vs. occipital), and Hemisphere (right vs. left) as within-subject factors, and Group (children vs. adults) as a between-subject factor, was performed. This analysis was followed by post hoc comparisons with Bonferroni corrections.

## 3. Results

### 3.1. Behavioral Results

The ANOVA with the within-subject factor Valence (negative vs. positive vs. neutral) and between-subject factor Group (children vs. adults) yielded a significant main effect of Valence, F(2,108) = 76.53, *p* < 0.001, partial *η*^2^ = 0.58. Post hoc comparisons showed that negative pictures (Mean = 5.0, SE = 0.18) were recalled better than positive (M = 4.44, SE = 0.18), *t*(108) = 3.62, *p*_bonf_ < 0.001, and neutral pictures (M = 3.13, SE = 0.18), *t*(108) = 12.06, *p*_bonf_ < 0.001. Positive pictures were also recalled better than neutral pictures, t(108) = 8.43, *p*_bonf_ < 0.001. A significant main effect of Group also emerged, F(1, 54) = 56.25, *p* < 0.001, partial *η*^2^ = 0.51, with the adults (M = 5.32, SE = 0.21) performing better than the children (M = 3.06, SE = 0.21).

**Figure 3:**
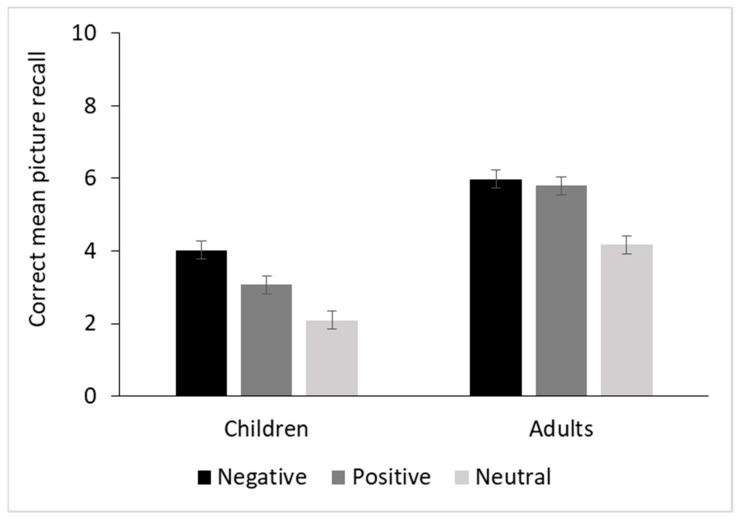
Mean correct responses for free recall of pictures as a function of group (children vs. adults) and emotional valence (negative vs. positive vs. neutral). Maximum score: 10 for each category of valence. Error bars represent standard errors.

Moreover, a significant Valence x Group interaction emerged, F(2, 108) = 3.66, *p* < 0.03, partial *η*^2^ = 0.07. Post hoc comparisons showed that adults had better recall for negative (M = 5.98, SE = 0.25, *t*(108) = 8.6, *p*_bonf_ < 0.001), and positive (M = 5.80, SE = 0.25, *t*(108) = 7.8, *p*_bonf_ < 0.001) than for neutral pictures (M = 4.17, SE = 0.25). The difference between negative and positive pictures was not significant, *t*(108) = 0.8, *p*_bonf_ = 1.0. Children had better recall for negative pictures (M = 4.02, SE = 0.25) than for positive (M = 3.07, SE = 0.25, *t*(108) = 4.2, *p*_bonf_ < 0.001) and neutral pictures (M = 2.09, SE = 0.25, *t*(108) = 8.5, *p*_bonf_ < 0.001). Positive pictures were also recalled better than neutral ones, *t*(108) = 4.3, *p*_bonf_ < 0.001 (see Figure 3).

The interaction Block x Group was also significant, F(5, 270) = 3.27, *p* < 0.007, partial *η*^2^ = 0.06. Post hoc comparisons showed that adults had similar performances across all blocks (all *p*_bonf_ > 0.54) and children had significantly better performances for the first two blocks than the sixth block (all *p*_bonf_ < 0.001). The interactions of Block with other factors were not significant.

### 3.2. ERP Results

ERP results were divided into three sections: the *emotion effect*, *successful encoding effect,* and *Dm effect*. For each section, the global analysis for children and adults is presented first, followed by separate analyses for each group, where appropriate. For clarity of presentation of the results, only the main effects and interactions that inform the research question are reported. All the ERP results in the text and figures are expressed in microvolts (μV).

### 3.3. Emotion Effect

The global ANOVA yielded a significant main effect of Valence, F(2, 88) = 7.94, *p* < 0.001, partial *η*^2^ = 0.15. Post hoc comparisons showed that ERPs for negative (Mean = −2.38, SE = 0.39), *t*(88) = 3.74, *p*_bonf_ < 0.001, and positive pictures (M = −2.49, SE = 0.39), *t*(88) = 3.05, *p*_bonf_ < 0.009, were more positive than ERPs for neutral pictures (M = −2.98, SE = 0.39). The difference between the negative and positive pictures was not significant, t(88) = 0.69, p_bonf_ = 1.00. The Window × Group and Cluster × Group interactions were also significant, F(1.22, 53.70) = 21.14, *p* < 0.001, partial *η*^2^ = 0.33 and F(1.20, 52.89) = 14.68, *p* < 0.001, partial *η*^2^ = 0.25, respectively. Moreover, importantly, a significant Valence × Window × Group interaction, F(3.08, 135.63) = 4.88, *p* < 0.003, partial *η*^2^ = 0.10, and a trend for the Valence × Window × Cluster × Group interaction, F(4.26, 187.66) = 2.12, *p* = 0.08, partial *η*^2^ = 0.05, emerged. To better understand the latter interactions which are significant (Valence × Window × Group) and trendy (Valence × Window × Cluster × Group), we decided to perform the analyses for each group separately (see below).

The ANOVA realized separately for children yielded a significant main effect of Valence, F(2, 38) = 4.06, *p* < 0.03, partial *η*^2^ = 0.18. Post hoc comparisons showed that ERPs for negative pictures (M = −3.61, SE = 0.85) were more positive than ERPs for neutral pictures (M = −4.57, SE = 0.85), *t*(38) = 2.83, *p*_bonf_ < 0.03. The differences between the negative and positive pictures (M = −3.99, SE = 0.85), and between the positive and neutral pictures were not significant, *t*(38) = 1.13, *p*_bonf_ = 0.80, and *t*(38) = 1.70, *p*_bonf_ = 0.29, respectively.

**Figure 4:**
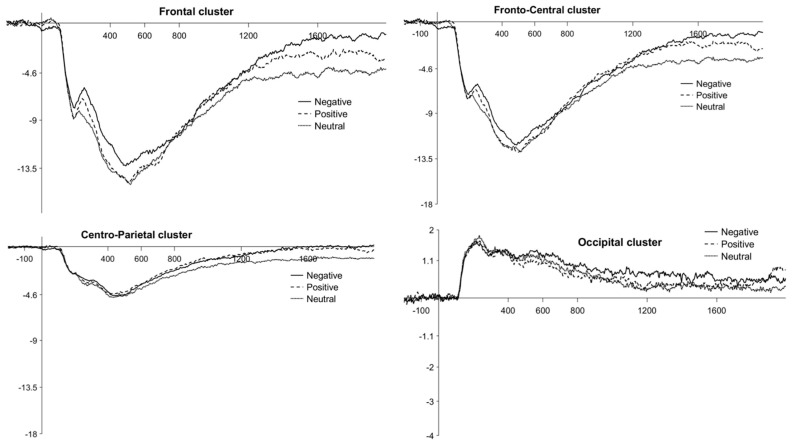
Children’s LPP waveforms for the *emotion effect* at the frontal, fronto-central, centro-parietal, and occipital clusters. Negative, positive and neutral conditions are plotted with different continuous or broken lines.

More precisely, a significant Valence × Window interaction, F(8, 152) = 4.28, *p* < 0.001, partial *η*^2^ = 0.18, a trend for Valence × Cluster interaction, F(6, 114) = 2.11, *p* = 0.057, partial *η*^2^ = 0.10, and a significant Valence × Window × Cluster interaction, F(24, 456) = 2.36, *p* < 0.001, partial *η*^2^ = 0.11, also emerged. Post hoc comparisons for the latter interaction showed that ERPs for negative pictures were more positive than for neutral pictures only at the frontal cluster during the W4 (1200–1600 ms) (negative: M = −2.45, SE = 1.21; neutral: M = −4.92, SE = 1.21), *t*(456) = 4.62, *p*_bonf_ < 0.02, and the W5 (1600–2000 ms) time windows (negative: M = −1.26, SE = 1.21; neutral: M = −4.45, SE = 1.21), *t*(456) = 5.96, *p*_bonf_ < 0.001, and at the fronto-central cluster during the W5 time window (negative: M = −1.32, SE = 1.21; neutral: M = −3.69, SE = 1.21), *t*(456) = 4.43, *p*_bonf_ < 0.03. There were no other significant main effects nor interactions of interest. Waveforms at the frontal, fronto-central, centro-parietal, and occipital clusters are plotted in Figure 4. Descriptive statistics are reported in Table 3.

**Figure 5:**
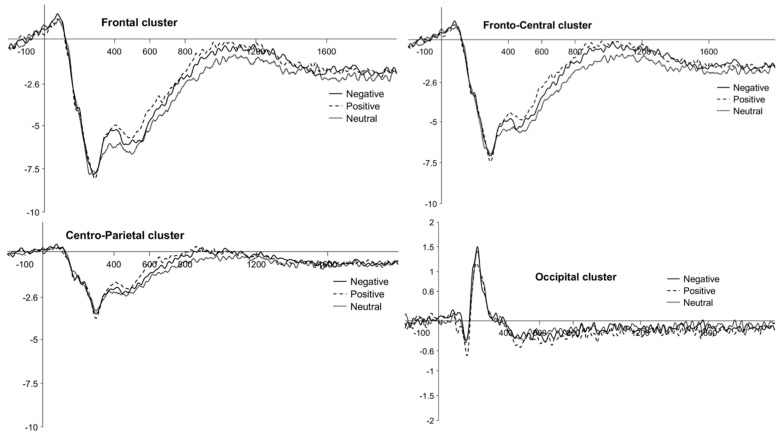
Adults’ LPP waveforms for the *emotion effect* at the frontal, fronto-central, centro-parietal, and occipital clusters. Negative, positive and neutral conditions are plotted with different continuous or broken lines.

The ANOVA realized separately for adults yielded a significant main effect of Valence, F(1.53, 38.37) = 6.93, *p* < 0.005, partial *η*^2^ = 0.22. Post hoc comparisons showed that ERPs for positive pictures (M = −1.32, SE = 0.21) were more positive than ERPs for neutral pictures (M = −1.73, SE = 0.21), *t*(50) = 3.70, *p*_bonf_ < 0.002. A trend toward ERPs being more positive for negative pictures (M = −1.48, SE = 0.21) than for neutral pictures also emerged, *t*(50) = 2.24, *p*_bonf_ = 0.09. The difference between positive and negative pictures was not significant, *t*(50) = 1.46, *p*_bonf_ = 0.45. More precisely, a significant Valence × Cluster interaction, F(3.10, 77.59) = 4.80, *p* < 0.004, partial *η*^2^ = 0.16, and Valence × Hemisphere interaction, F(1.92, 48) = 3.45, *p* < 0.04, partial *η*^2^ = 0.12, emerged. Post hoc comparisons for the Valence × Cluster interaction showed that ERPs were more positive for positive pictures than for neutral pictures at the frontal (positive: M = −2.52, SE = 0.27; neutral: M = −3.09, SE = 0.27), *t*(136.8) = 3.80, *p*_bonf_ < 0.02, and fronto-central clusters (positive: M = −1.92, SE = 0.27; neutral: M = −2.59, SE = 0.27), *t*(136.8) = 4.48, *p*_bonf_ < 0.002, with a trend at the centro-parietal cluster (positive: M = −0.56, SE = 0.27; neutral: M = −1.07, SE = 0.27), *t*(136.8) = 3.41, *p*_bonf_ = 0.057. Moreover, post hoc comparisons for the Valence × Hemisphere interaction showed that ERPs for positive pictures were more positive than for neutral pictures in the left hemisphere only (positive: M = −1.30, SE = 0.22; neutral: M = −1.81, SE = 0.22), *t*(62.1) = 4.35, *p*_bonf_ < 0.001. There were no other significant main effects nor interactions of interest. Waveforms at the frontal, fronto-central, centro-parietal, and occipital clusters are plotted in Figure 5. Descriptive statistics are reported in Table 4.

### 3.4. Successful Encoding Effect

The global ANOVA yielded a significant main effect of Recall, F(2, 40) = 7.11, *p* < 0.01, partial *η*^2^ = 0.15, with ERPs being more positive for recalled pictures (M = −2.30, SE = 0.36) than for forgotten pictures (M = −2.58, SE = 0.36). The Recall × Cluster interaction was also significant, F(1.39, 55.51) = 8.11, *p* < 0.003, partial *η*^2^ = 0.17. Post hoc comparisons showed that ERPs were more positive for recalled than forgotten pictures at the frontal (recalled: M = −4.71, SE = 0.47; forgotten: M = −5.18, SE = 0.47), *t*(96.7) = 3.51, *p*_bonf_ < 0.02, and fronto-central clusters (recalled: M = −3.70, SE = 0.47; forgotten: M = −4.20, SE = 0.47), *t*(96.7) = 3.71, *p*_bonf_ < 0.01. Moreover, a significant Valence x Recall × Window × Cluster × Group interaction, F(5.87, 234.79) = 2.92, *p* < 0.01, partial *η*^2^ = 0.07, emerged. To better understand the latter Valence × Recall × Window × Cluster × Group interaction, we decided to perform the analysis for each group separately (see below). There were no other significant main effects nor interactions of interest.

**Figure 6:**
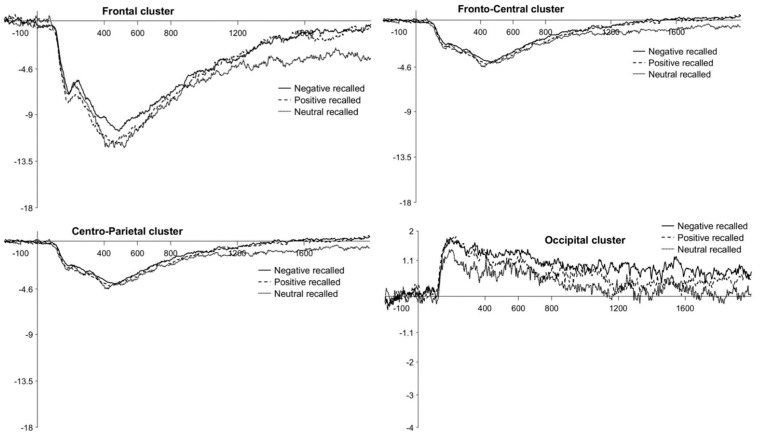
Children’s LPP waveforms for the *successful encoding effect* at the frontal, fronto-central, centro-parietal, and occipital clusters. Negative, positive, and neutral conditions are plotted with different continuous or broken lines.

The ANOVA realized separately for children showed only one significant interaction for Valence × Recall × Window × Cluster, F(24, 360) = 2.42, *p* < 0.001, partial *η*^2^ = 0.14. Post hoc comparisons showed that ERPs were more positive for negative recalled than neutral recalled pictures during the W5 time window at the frontal cluster (negative: M = −0.69, SE = 1.34; neutral: M = −5.09, SE = 1.34), *t*(213.2) = 5.60, *p*_bonf_ < 0.001. Moreover, during the same time window and at the same cluster, a trend toward more positive ERPs for positive recalled (M = −1.60, SE = 1.34) than neutral recalled pictures was also observed, *t*(213.2) = 4.44, *p*_bonf_ = 0.09. There were no other significant main effects nor interactions of interest. Waveforms at the frontal, fronto-central, centro-parietal, and occipital clusters are plotted in Figure 6. Descriptive statistics are reported in Table 5.

**Figure 7:**
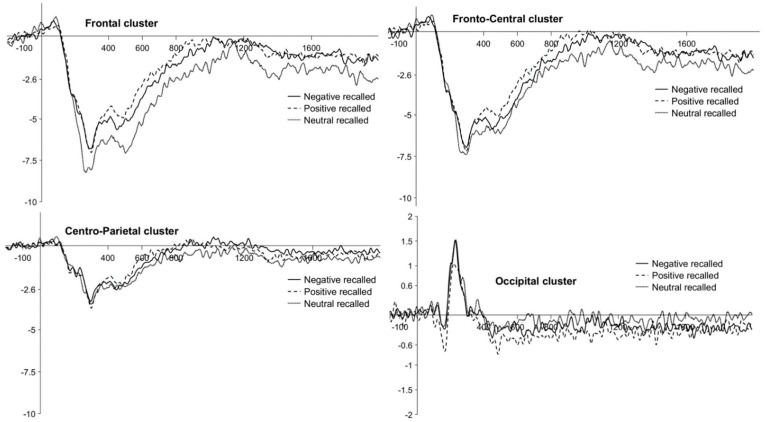
Adults’ LPP waveforms for the *successful encoding effect* at the frontal, fronto-central, centro-parietal, and occipital clusters. Negative, positive and neutral conditions are plotted with different continuous or broken lines.

The ANOVA realized separately for adults yielded a significant main effect of Recall, F(1, 25) = 4.24, *p* < 0.05, partial *η*^2^ = 0.15, with ERPs being more positive for recalled pictures (M = −1.48, SE = 0.21) than for forgotten pictures (M = −1.65, SE = 0.21). Moreover, a trend emerged for the Valence × Recall × Cluster interaction, F(2.63, 65.69) = 2.33, *p* = 0.09, partial *η*^2^ = 0.09. Post hoc comparisons for the latter interaction showed that ERPs were more positive for positive recalled than for neutral recalled pictures at the frontal (positive: M = −2.39, SE = 0.29; neutral: M = −3.13, SE = 0.29), *t*(258) = 4.42, *p*_bonf_ < 0.004, and fronto-central clusters (positive: M = −1.75, SE = 0.29; neutral: M = −2.61, SE = 0.29), *t*(258) = 5.13, *p*_bonf_ < 0.001. There were no other significant main effects nor interactions of interest. Waveforms at the frontal, fronto-central, centro-parietal, and occipital clusters are plotted in Figure 7. Descriptive statistics are reported in Table 6.

### 3.5. Dm Effect

The global ANOVA did not show any significant main effects nor interactions (all *p* > 0.05).

## 4. Discussion

In the present study, we investigated the effect of emotions on episodic memory in 8- to 11-year-old children and young adults, using behavioral and ERP measures. To do this, all participants performed pictures’ intentional encoding tasks while ERPs were recorded, followed by immediate free-recall tasks.

### 4.1. Behavioral Results

According to our prediction, we observed an EEM in both groups with better recall of negative and positive pictures relative to neutral ones. Moreover, a *negativity effect* occurred in children but not in adults, with negative pictures being recalled even better than positive ones. These results are consistent with other studies that have shown that the emotional content of information enhances episodic memory in children (for a review, see [30]) and adults (for a review, see [2]). The *negativity effect* observed in children is also in line with other studies who showed better memory for negative and positive stimuli with an advantage for negative ones in 5- to 6-year-old [25] and 8- to 11-year-old children [26]. However, we did not observe a *negativity effect* in recall performances for adults. Nevertheless, this effect is not systematic in the adult literature, as it may vary, at least in part, based on factors such as the type of stimuli used and/or how relevant they are to one’s goals [44]. Overall, our results also showed better recall performances in adults compared to children, suggesting that episodic memory processes in school-age children are still developing [45]. Better performances for the first two blocks compared to the sixth block have also emerged in children. As the blocks were balanced between the participants, this difference cannot be related to the pictures within the blocks, but may be due to the mental fatigue of children over time which could affect memory performance, as mental fatigue is known to affect cognitive performance in general [46,47].

### 4.2. ERP Results—Emotion Effect

With regard to the ERP responses, the global analysis showed an *emotion effect* characterized by a greater modulation of the LPP during the encoding of emotional pictures, negative and positive, relative to neutral ones in both groups. Additional analyses showed that children exhibited an *emotion effect* with a more positive amplitude of the LPP especially for negative pictures compared to neutral ones during the W4 and W5 time windows, which is 1200 until 2000 ms after the stimulus onset, at the frontal and fronto-central clusters. In adults, however, a greater amplitude of the LPP was observed across all time windows especially for positive pictures relative to neutral ones at the frontal and fronto-central clusters and a tendency was observed at the centro-parietal cluster. Moreover, this *positivity effect* was lateralized in the left hemisphere only. These results, showing that children were more sensitive to negative pictures and adults to positive ones, are only partly consistent with our predictions that both positive and negative pictures would elicit greater LPP amplitude compared to neutral ones in children and adults.

Concerning children, the *emotion effect* observed for negative pictures in our study was also found by the Ref. [29]. Notably, the authors highlighted greater LPP responses in 7.5- to 9-year-old children during the encoding of negative and positive pictures than neutral ones from 800 to 2000 ms after stimulus onset, at the posterior cluster for the former and at the frontal and central clusters for the latter. Although the timing of the *emotion effect* is consistent between their study and ours, we did not observe this negative/posterior and positive/fronto-central dissociation in our study since only negative pictures elicited larger LPP at anterior clusters. These discrepancies could be due in part to developmental changes that occur at the neural level for emotion processing, as children in our study were, on average, older than the children in the study by the Ref. [29]. Indeed, some other ERP studies that have investigated the development of emotion-processing, albeit without memory tasks [19,34,35], offer some evidence of a shift in the scalp distribution of the LPP from posterior sites in middle childhood to more centro-parietal regions in adolescence [19,34,35]. In these studies, both negative and positive pictures were associated with a larger LPP compared to neutral pictures, and negative pictures also elicited larger LPP compared to positive pictures, for all groups of age from 8- to 15-year-old children. However, neither of these studies analyzed frontal sites, although we know that the LPP may be sustained by frontal networks in adulthood [48]. Therefore, in our study, it is likely that the *emotion effect* observed at the frontal sites in 8- to 11-year-old was due to a relatively more advanced development of negative emotion-processing. It is noteworthy that this finding cannot be related to the arousal of the pictures which were identical between the negative and positive ones according to the normative children’s ratings of arousal. However, it could be partly due to the greater attentional bias toward negative pictures reported in school-age children ([49], but for a review, see [50]), which can lead to these stimuli being processed more thoroughly during encoding and this, in turn, would enhance their neural responses and their subsequent memorability (for a review, see [51]). Other explanations for this *negativity effect* could be that positive stimuli may be less consistent as for the emotion they generate in the participants, which means that some positive pictures may not be perceived as such or less strongly for some participants. Moreover, some work indicates that emotions could enhance memory for information relevant to the subject’s current motivational state (for a review, see [52]). Thus, interindividual differences may potentially account for this lack of effect for positive pictures in ERP responses in our study, especially since a recent study by the Ref. [53] has shown that interindividual differences can predict how one will perceive and respond to emotional information.

Concerning adults, greater LPP during the encoding of positive pictures and a trend toward greater LPP during the encoding of negative pictures were observed compared to neutral ones, even though only a *positivity effect* appeared at anterior clusters and in the left hemisphere after more detailed analysis. These ERP results, associated with a general EEM in free recall performances, are consistent with an ERP study in which general EEM was observed in free recall in adults associated with a larger LPP during the encoding of positive pictures compared to negative and neutral ones at frontal site, albeit with a general *emotion effect* over parietal site [9]. Moreover, the authors also observed a larger LPP for positive pictures in the frontal left hemisphere during the 500–800 ms epoch as compared with the right hemisphere. Together with our findings, these results support the lateralized valence discrimination effect [54].

From a developmental perspective, our study revealed some discrepancies between children and adults concerning the lateralization and the occurrence of the *emotion effect*. Indeed, this effect was lateralized in the left hemisphere for adults while there was no difference between the hemispheres for the children, and occurred throughout the presentation of the stimulus for adults and only during the late time-windows, from 1200 to 2000 ms after stimulus onset, for children. These differences between children and adults suggest that emotion processes are still developing in school-age children [55]. Moreover, the late modulation of the LPP in children may be related to the immaturity of some cognitive processes, such as the speed and/or efficiency of encoding [20], as well as the progressive development of the frontal cortex into adulthood that partly supports these processes (e.g., [56]).

### 4.3. ERP Results—Successful Encoding and Dm Effects

In order to investigate the neural correlates underlying emotional memory in children and adults, we analyzed the ERP responses during the successful encoding of pictures (i.e., pictures that were later recalled). The global analysis showed that both groups had a more positive amplitude of the LPP for subsequently recalled than for forgotten pictures overall, mostly at the frontal and fronto-central clusters. These findings are consistent with some studies that showed that the amount of activity in the prefrontal cortex (PFC) during encoding relates to the likelihood that information is later remembered, in relation to controlled encoding processes (for a review, see [57]). Specifically, separate analyses for both groups showed that children exhibited greater LPP for negative recalled than for neutral recalled pictures, and a trend toward greater LPP for positive recalled than for neutral recalled pictures, during the W5 time window at the frontal cluster. These ERP responses suggest that neural responses during encoding can predict behavioral outcomes. Therefore, the later-emerging attentional processes at anterior sites might be most closely implicated in the interaction between emotion processes and episodic memory in children as seen with the late emergence of the LPP for successful encoding of emotional pictures. These findings suggest that the involvement of cognitive factors during encoding, such as attention, may support the EEM in children in the same way as in adults. This provides preliminary evidence for the relevance of the cognitive mediation hypothesis [7] in individuals still in a period of development.

The group of adults, however, had a greater LPP for positive recalled than neutral recalled pictures at the frontal and fronto-central clusters, across all time windows, although a general EEM was observed in their free recall performances. These results are consistent with some studies that showed greater engagement of the PFC in the encoding of information with positive rather than negative valence in adults (e.g., [58]).

Although the ERP responses for the successful encoding of pictures were modulated by emotions in children and adults, the *Dm effect* (pictures that were later recalled minus pictures that were later forgotten), however, was not significantly different between all the conditions (negative, positive, neutral) and between the two groups of participants. Therefore, these findings do not allow us to validate our hypothesis that children and adults would experience a larger *Dm effect* for emotional compared to neutral pictures. Yet, this effect has been already investigated with emotional pictures in adults [9] and the authors have shown larger *Dm effect* for negative and positive pictures compared to neutral ones during the 400–600 ms time window, and maximal at the midline central electrode. Unfortunately, to our knowledge, this effect has never been investigated with emotional pictures in children. Nevertheless, some ERP studies of memory without emotional stimuli have investigated this effect in both adults and children to identify potential developmental differences in the ERP responses associated with memory processes. Specifically, [59] have shown that the *Dm effect* was comparable among 6- to 8-year-old children, and 12- to 13-year-old adolescents and young adults, during the 700–900 ms time window at frontal, central, and parietal sites. Therefore, this study shows that not only can the *Dm effect* be measured in children, but that the neural responses related to memory encoding are already well-developed in children, as they are similar to those of adults. Hence, further studies are needed to investigate the *Dm effect* with emotional stimuli in children, in order to learn more about the changes in the neural correlates supporting emotional memory during development.

## 5. Conclusions and Limitations

The present study compared, for the first time, the EEM between school-age children and adults at both the behavioral and physiological levels, and brought out some similarities between the two groups. Indeed, both groups had better episodic memory for negative and positive pictures compared to neutral ones, with an advantage for negative over positive pictures for children only. The ERP responses, recorded during the encoding of pictures, showed that the *emotion effect* was localized mainly at the frontal and fronto-central clusters in children and adults, although it was more of a *negativity effect* for children and a *positivity effect* for adults. Moreover, the *emotion effect* occurred throughout the presentation of the stimulus for adults and only during the late time windows, from 1200 to 2000 ms after stimulus onset, for children. Further analyses showed that this *negativity* effect in children and *positivity* effect in adults was also apparent during the successful encoding of pictures. Indeed, larger neural responses were observed for the successful encoding of negative pictures in children and positive pictures in adults, compared to neutral ones, at anterior sites. Moreover, this effect was also delayed in children, as it appeared only during the late time window compared with adults for whom the effect was present for all time windows. Taken together, these findings suggest that the interaction between emotional and memory systems has already been established at the behavioral level in 8- to 11-year-old children, although some discrepancies between the two groups in ERP responses indicate that the neural correlates underlying EEM in children are probably still developing. Moreover, these findings provide a direct connection between the effect of emotions on memory observed in behavioral performance, and the greater attentional engagement toward emotional stimuli during encoding reflected by the modulation of the LPP, both in children and adults. Therefore, this study is the first to provide evidence of the relevance of the cognitive mediation hypothesis in children, and deserves to be further explored in future research. However, our study has an important limitation, that is, the sample size of the experimental groups, and thus our data must be taken with precaution. In future research, it would be necessary to increase the number of participants, and perhaps the number of stimuli presented, and/or to include groups with a smaller age range. Regarding the number of participants, unfortunately the health situation related to COVID-19 did not allow us to include more participants. However, a previous study on EEM in children [29] had significant effects in the ERP analysis in recognition task with only 15 participants included in the analysis, although this study had a less complex design. Other limitations are present in our study, such as the reduced amplitude of the EEG signal observed at posterior electrodes (parietal and occipital) in some of the participants. This may be responsible for a lack of effect on the posterior clusters in children and adults. In addition, participants were asked to indicate whether they saw a human face in the pictures during encoding. Although some studies have shown greater LPP for emotional faces compared to neutrals in children [60] and adults [43], it is possible that this task may have changed how participants process the emotional content of the pictures and hence, their neural responses to emotions. Moreover, there was a gender imbalance in our participant groups. As one study showed that gender could potentially affect the neural processing of emotions in children [33], future research should consider the role that gender can play in the neural responses associated with the EEM in children. In addition, it would also be interesting to investigate the role of emotion regulation in EEM in children and adults as to better understand its mechanisms [2]. Finally, more studies are needed to further investigate the EEM using multiple measures and different age groups of children in order to better understand the effect of emotions on episodic memory throughout development, and make a stronger connection with the real-world experiences of emotion.

## Figures and Tables

**Table 1:** Mood, handedness, and cognitive functioning assessments for children and adults. Note: Raw mean scores are presented for FMATC, BMIS, and Edinburgh Inventory. Mean percentile ranks are presented for subtests of WISC V. Standard deviations are in parentheses.

Function	Test	Children *n* = 26	Adults *n* = 30
	Mean Age	9.83 (1.45)	21.47 (2.47)
	Female/Male	10 girls and 16 boys	16 women and 14 men
Mood	FMATC BMIS	5.38 (0.57) 41.12 (3.48)	/ 52.17 (4.40)
Handedness	Edinburgh Inventory	80.54 (21.40)	80.10 (20.05)
Cognitive functioning	WISC-V Similarities WISC-V Matrix Reasoning	0.70 (0.21) 0.73 (0.18)	/ /

**Table 2:** Mean number of epochs included in ERP averages for the *Dm effect* for children and adults. Standard deviations are in parentheses.

	Children (*n* = 16)		Adults (*n* = 26)	
Pictures	Remembered	Forgotten	Remembered	Forgotten
Negative	26.44 (7.81)	27.19 (7.07)	34.35 (7.83)	23.65 (8.00)
Positive	18.06 (5.05)	34.63 (7.68)	32.50 (6.89)	25.35 (6.58)
Neutral	13.75 (8.87)	38.69 (9.69)	22.00 (6.84)	34.42 (6.95)

**Table 3:** Children–Waveforms–Emotion effect. ^a^ 95% CI for Mean Difference, ^b^ Cohen’s d does not correct for multiple comparisons. The significant comparisons are indicated by *. F: Frontal; FC: Fronto-Central; W: Window. For interactions, only significant or trend data are reported.

Variables	Mean Difference (SE)	*p*_bonferroni_	Effect Size (Cohen’s d) ^b^	CI Lower ^a^	CI Upper ^a^
Valence					
Negative vs. Positive	0.383 (0.340)	0.799	0.252	−0.467	1.234
Negative vs. Neutral	0.961 (0.340)	0.022 *	0.633	0.110	1.812
Positive vs. Neutral	0.578 (0.340)	0.291	0.380	−0.273	1.428
Valence × Window × Cluster					
F W4: Neg vs. Neu	2.474 (0.535)	0.012 *	/	0.177	4.771
F W5: Neg vs. Neu	3.189 (0.535)	<0.001 *	/	0.892	5.486
FC W5: Neg vs. Neu	2.373 (0.535)	0.028 *	/	0.076	4.670

**Table 4:** Adults–Waveforms–Emotion effect. ^a^ 95% CI for Mean Difference, ^b^ Cohen’s d does not correct for multiple comparisons. The significant comparisons are indicated by *. F: Frontal; FC: Fronto-Central; CP: Centro-Parietal; LH: Left Hemisphere. For interactions, only significant or trend data are reported.

Variables	Mean Difference (SE)	*p*_bonferroni_	Effect Size (Cohen’s d) ^b^	CI Lower ^a^	CI Upper ^a^
Valence					
Negative vs. Positive	0.161 (0.110)	0.452	0.286	−0.434	0.112
Negative vs. Neutral	0.246 (0.110)	0.090	0.439	−0.027	0.519
Positive vs. Neutral	0.407 (0.110)	0.002 *	0.725	0.134	0.680
Valence × Cluster					
F: Pos vs. Neu	0.568 (0.149)	0.014 *	/	0.053	1.083
FC: Pos vs. Neu	0.669 (0.149)	0.001 *	/	0.155	1.184
CP: Pos vs. Neu	0.509 (0.149)	0.057	/	−0.006	1.023
Valence × Hemisphere					
LH: Pos vs. Neu	0.508 (0.117)	<0.001 *	/	0.151	0.864

**Table 5:** Children–Waveforms–Successful encoding effect. ^a^ 95% CI for Mean Difference, ^b^ Cohen’s d does not correct for multiple comparisons. The significant comparisons are indicated by *. F: Frontal; W: Window; Rec: Recalled. For interactions, only significant or trend data are reported.

Variables	Mean Difference (SE)	*p*_bonferroni_	Effect Size (Cohen’s d) ^b^	CI Lower ^a^	CI Upper ^a^
Valence × Recall × Window × Cluster					
F W5 Rec: Neg vs. Neu	4.400 (0.79)	<0.001 *	/	0.782	8.017
F W5 Rec: Pos vs. Neu	3.487 (0.79)	0.09	/	−0.130	7.105

**Table 6:** Adults—Waveforms—Successful encoding effect. ^a^ 95% CI for Mean Difference, ^b^ Cohen’s d does not correct for multiple comparisons. The significant comparisons are indicated by *. F: Frontal; FC: Fronto-Central; Rec: Recalled. For interactions, only significant or trend data are reported.

Variables	Mean Difference (SE)	p_bonferroni_	Effect Size (Cohen’s d) ^b^	CI Lower ^a^	CI Upper ^a^
Recall					
Recalled vs. Forgotten	0.162 (0.08)	0.05 *	0.404	0.000	0.324
Valence × Recall × Cluster					
F Rec: Pos vs. Neu	0.738 (0.17)	0.004 *	/	0.104	1.373
FC Rec: Pos vs. Neu	0.857 (0.17)	<0.001 *	/	0.222	1.491

## Data Availability

The data and material for the experiment may be available on request. The experiment was not preregistered.

## Appendix A

**Table A1:** Mean ratings of emotional valence, arousal and visual complexity of pictures from the DAPS database, for children and adults. Standard deviations are in parentheses. For both groups, the negative and positive pictures did not overlap with the neutral pictures in terms of normative valence and arousal ratings. ^a^ Valence and arousal are rated on a scale from 1 (very negative/not arousing at all) to 9 (very positive/highly arousing). ^b^ Visual complexity is rated on a scale from 1 (very simple) to 4 (very complex).

APS Database	Children			Adults		
Pictures	Valence ^a^	Arousal ^a^	Complexity ^b^	Valence ^a^	Arousal ^a^	Complexity ^b^
Negative	3.21 (0.34)	6.12 (0.90)	2.53 (0.53)	3.21 (0.28)	5.92 (0.43)	2.57 (0.52)
Positive	7.41 (0.42)	6.19 (0.71)	2.47 (0.52)	7.04 (0.34)	5.77 (0.41)	2.41 (0.63)
Neutral	5.93 (0.42)	3.86 (0.52)	2.38 (0.47)	5.62 (0.39)	3.61 (0.55)	2.28 (0.47)
